# Cystatin C, Ammonia, and Bicarbonate Measurements in the Saliva of Pigs: Analytical Validation and Changes in *S. suis* Infection

**DOI:** 10.3390/ani14111580

**Published:** 2024-05-27

**Authors:** Eva Llamas-Amor, Elena Goyena, Antonio González-Bulnes, Edgar García Manzanilla, José Joaquín Cerón, Silvia Martínez-Subiela, María José López-Martínez, Alberto Muñoz-Prieto

**Affiliations:** 1 Interdisciplinary Laboratory of Clinical Analysis (Interlab-UMU), Regional Campus of International Excellence ‘Campus Mare Nostrum’, University of Murcia, Campus de Espinardo, 30100 Murcia, Spain; eva.llamasa@um.es (E.L.-A.); jjceron@um.es (J.J.C.); silviams@um.es (S.M.-S.); alberto.munoz@um.es (A.M.-P.); 2 Department of Animal Health, Faculty of Veterinary Medicine, University of Murcia, 30100 Murcia, Spain; goyena@um.es; 3 Departamento de Producción y Sanidad Animal, Facultad de Veterinaria, Universidad Cardenal Herrera-CEU, CEU Universities, C/Tirant lo Blanc, 7, 46115 Alfara del Patriarca, Spain; antoniogbulnes@cuartesa.com; 4 Cuarte S.L. Grupo Jorge, Ctra. De Logroño, Km 9.2, Monzalbarba, 50120 Zaragoza, Spain; 5 Pig Development Department, The Irish Food and Agriculture Authority, Teagasc, P61 C996 Cork, Ireland; egmanzanilla@gmail.com; 6 School of Veterinary Medicine, University College Dublin, D04 V1W8 Dublin, Ireland

**Keywords:** cystatin C, ammonia, bicarbonate, automated assay, pig, sepsis

## Abstract

**Simple Summary:**

The use of saliva as a sample for the measurements of biomarkers is growing in pigs due to its easy collection, which does not produce stress in animals. Cystatin C, ammonia, and bicarbonate have been reported to be biomarkers of sepsis and inflammation in humans, but there is a lack of information about assays for the measurement of these analytes in pigs. The objective of this report was to validate commercially available assays for the measurement of Cystatin C, ammonia, and bicarbonate in the saliva of pigs and study the variations of these analytes in a septic disease such as Streptococcus suis (*S. suis*) infection. The assays showed adequate precision and accuracy for the measurements of the three analytes in the saliva of pigs. In addition, −80 °C was the temperature recommended to store samples for the measurement of these analytes. Pigs with *S. suis* infection have higher mean concentrations of cystatin and ammonia and lower concentrations of bicarbonate in their saliva than healthy pigs. Based on these results, cystatin C, ammonia, and bicarbonate can be measured in the saliva of pigs and can be potential biomarkers in this species.

**Abstract:**

Cystatin C, ammonia, and bicarbonate have been described to be biomarkers of sepsis and inflammation in humans. The saliva of pigs can be used to detect a wide range of pathogens but also many biomarkers that can be analyzed to evaluate different conditions such as stress (i.e., cortisol and alpha amylase), immune system (i.e., ADA, S100 proteins), inflammation (i.e., acute phase proteins), redox status (i.e., various antioxidants and oxidants), and general metabolism or the status of different organs and tissues. However, there is a lack of assays for the possible measurement and use of cystatin C, ammonia, and bicarbonate in saliva as biomarkers of sepsis or inflammation in pigs. The objective of this study was to validate commercially available automated assays for the measurement of cystatin C, ammonia, and bicarbonate in the saliva of pigs, having the advantage of using a noninvasive sample that is easy to collect. The assays were precise and accurate, and the recommended storage condition for the saliva samples was −80 °C. In addition, cystatin and ammonia showed significant increases in the saliva of pigs with *S. suis* infection, whereas bicarbonate decreased. Further studies would be recommended to increase knowledge about the possible potential applications of the measurements of these three analytes in the saliva of pigs as biomarkers to evaluate the animals’ health and welfare.

## 1. Introduction

Saliva is increasingly used as a sample for measuring biomarkers in humans [1,2] and animals [3,4]. In particular, in pigs, saliva collection is more convenient than blood collection since it is simpler, faster, cheaper, and easy to perform, producing less stress for the animals [1,5,6]. In addition, there is no need for specialized personnel for sampling, which makes saliva an ideal specimen for being collected in routine farm conditions. Furthermore, it is very convenient and useful for monitoring conditions in which various samplings are required in a relatively short time. Overall, using saliva as a sample can significantly improve animal health and welfare [6,7,8].

In the saliva of pigs can be detected a wide range of pathogens but also many biomarkers that can be used to evaluate different conditions such as stress (i.e., cortisol and alpha-amylase), immune system (i.e., ADA, S100 proteins), inflammation (i.e., acute phase proteins), redox status (i.e., various antioxidants and oxidants), and general metabolism or the status of different organs and tissues [1,6,7]. In addition, recently, biomarkers of sepsis, such as procalcitonin and aldolase, have been described [9,10]. However, there is a lack of information about the analysis and use of other possible biomarkers of sepsis, such as Cystatin C, ammonia, and bicarbonate, in the saliva of pigs. These markers can also be relevant in diagnosing and monitoring other diseases and conditions, particularly those involving renal function, liver function, and metabolic or acid-base imbalances.

Cystatin C has been traditionally considered a biomarker of kidney disease. However, interestingly, it is not only a good index of kidney functions but is also involved in immune regulation and apoptosis under various pathological conditions related to inflammation and immune system alterations [11]. In the saliva of pigs, increases in this protein in an experimentally induced sepsis and also in inflammation have been detected in a previous proteomic study [10].

Ammonia is also an analyte related to sepsis and inflammation. Sepsis causes swelling of intestinal mucosa, leading to defective barrier function and intestinal bacterial translocation, which produces additional ammonia in the intestine to be absorbed into the blood. In addition, in sepsis, there is an increase in metabolism and degradation of amino acids, while ammonia production appears to increase [12]. The increase in ammonia in sepsis can lead to encephalopathy, and it is recommended that serum ammonia levels be closely monitored in patients with sepsis [13]. To our knowledge, ammonia has not been evaluated in the saliva of sepsis or the saliva of pigs, but this is an analyte that can be measured in the saliva of humans and horses [14].

A decrease in serum bicarbonate was described in sepsis in humans [15] and ovine [16], possibly due to the metabolic acidosis associated with this condition. In addition, serum bicarbonate is a good predictor of clinical outcomes in sepsis alone or combined with other markers [17]. Bicarbonate has been measured in the saliva of pigs, giving similar values to that in humans [18]. Recently, changes in concentrations of ammonia and bicarbonate were detected in horses with gastric ulcers [14]. However, to the author`s knowledge, there is no analytical validation data of the measurement of bicarbonate in the saliva of pigs.

This manuscript hypothesizes that cystatin C, ammonia, and bicarbonate can be determined in the saliva of pigs with assays that could provide adequate analytical validation results and also that these analytes could change in saliva in a situation of sepsis, such as occurs in pigs with spontaneous *S. suis* infection. Therefore, the objectives of this study were to perform an analytical validation of commercially available automated assays for the measurements of cystatin C, ammonia, and bicarbonate in saliva and evaluate the possible changes in these analytes in pigs with *S. suis* infection.

## 2. Materials and Methods

### 2.1. Animals

This study involved weaning pigs aged 4 to 8 weeks, bred from a cross of Pietrain, Large White, and Landrace and sourced from a commercial farm in Spain. The pigs were housed in pens equipped with a standard feeder and nipple drinker, ensuring unrestricted access to food and water, in accordance with regulations [19]. Each pig was allocated a minimum space of 0.65 m^2^, and the ambient temperature was maintained at an average of 23 ± 2 °C.

Two different groups were made: (1) the healthy group (*n* = 33), integrated by clinically healthy pigs that did not show any external clinical sign at physical examination, and (2) the meningitis group (*n* = 27), integrated by pigs naturally infected by *S. suis*.

The established criteria for selecting pigs for the meningitis group were as follows: (1) displaying clinical symptoms consistent with *S. suis* infection, (2) having no previous treatment, and (3) testing positive for *S. suis* during bacterial isolation and characterization as was recommended for this pathogen [20]. To achieve this, blood samples were cultured on Columbia blood agar plates (Oxoid Ltd., Madrid, Spain) supplemented with 5% defibrinated pig blood for 48 h at 37 °C under aerobic conditions [21]. Identification of isolates was carried out using standard methods and confirmed via polymerase chain reaction (PCR) targeting the glutamate dehydrogenase gene, as previously outlined [22]. All the pigs in the meningitis group tested positive for *S. suis* serotype 9. Following diagnosis, the pigs were treated with injectable amoxicillin (Unicillin, Univet Ltd., Co., Cavan, Ireland). At the time, the pig farms did not have any metaphylactic treatment protocols in place.

All the pigs included were at 3 points (moderate) of the scale of disease severity, which was assessed using a five-point scale as follows: (1) no clinical signs (control group), (2) mild, (3) moderate, (4) high, and (5) severe disease. This classification has been determined based on the presence of hyperthermia, arthritis, and two levels of neurological symptoms, as per the criteria established in the prior publication [23].

### 2.2. Saliva Sampling

Saliva collection from the pigs was performed using a metal rod equipped with a sponge inserted into their mouths. Subsequently, the sponges were removed and placed into Salivette tubes (manufactured by Sarstedt, Aktiengesellschaft & Co., Nümbrecht, Germany). All the samples were promptly stored in a portable refrigerator at temperatures in the range of 4–8 °C until they reached the laboratory. Upon arrival, the saliva samples were placed into Eppendorf tubes and preserved at −80 °C until analysis. To separate the saliva supernatant, the Salivette tubes were submitted to centrifugation at 3000× *g* and 4 °C for 10 min. The resulting aliquots were then dispensed into the Eppendorf tubes and stored at −80 °C until further analysis.

In the case of the sick pig group, the saliva samples were collected prior to the administration of any treatment.

### 2.3. Biochemical Analysis of Saliva

Cystatin C was analyzed using an immunoturbidimetric assay using latex particles coated with an antibody against human cystatin C (Gentian Diagnostics ASA, Moss, Norway). 

Ammonia levels were measured using an enzymatic colorimetric assay (Spinreact, Girona, Spain). This method involves the reaction of ammonia with α-ketoglutarate and NADPH, catalyzed by glutamate dehydrogenase (GLDH), forming glutamate and NADP+. The decrease in absorbance at 340 nm corresponds to the plasma ammonia concentration.

Bicarbonate levels were quantified using an enzymatic colorimetric assay (Biosystem S.A., Barcelona, Spain). This assay relies on the consumption of the NADH cofactor by the carbon dioxide (CO_2_) present in the sample.

All three assays were adapted for use with an automated biochemical analyzer, the Olympus AU400 (Beckman Coulter, Brea, CA, USA).

### 2.4. Validation Study of the Assays

The assays were validated in the pig saliva samples using aliquots of pig saliva with different values of each analyte.

The validation of the assays was assessed as follows:

- Precision was assessed by calculating the intra- and inter-assay coefficients of variation (CVs) using saliva samples containing high and low cystatin C, ammonia, and bicarbonate concentrations.
- Accuracy evaluation was indirectly conducted by linearity after dilution studies using ultrapure water on saliva samples with elevated levels of ammonia and bicarbonate. In the case of cystatin C, interference with serial dilutions was observed, and a recovery study was performed, wherein samples with two different concentrations of the analyte were mixed at different concentrations.
- The lower limit of quantification (LLoQ) was calculated as the lowest concentration of Cystatin C, ammonia, and bicarbonate that the assays could determine with an intra-assay CV < 20%.
- The limit of detection (LD) was defined as the lowest concentration at which the assays could reliably distinguish a specimen with a zero value (ultrapure water). This determination was made by calculating the mean value plus three standard deviations from twelve replicate measurements of ultrapure water.

### 2.5. Stability Study

The influence of different storage temperatures over time was examined to evaluate the determination of cystatin C, ammonia, and bicarbonate levels in the pig saliva.

Five saliva samples were collected from five pigs at the Teaching Farm of the University of Murcia, and measurements were taken at various time points. Initially (T0), each sample was divided into four subsamples: one stored at room temperature, one refrigerated at 4 °C, one frozen at −20 °C, and one frozen at −80 °C. Concentrations of cystatin C, ammonia, and bicarbonate were assessed before storage (T0) and after 24 and 48 h.

### 2.6. Cystatin C, Ammonia, and Bicarbonate Concentrations in the Saliva of Pigs Infected with S. suis

The changes in the analyte concentrations were compared in the different groups of pigs included in the study and are described in Section 2.1.

In this trial, the saliva samples collected from all the pigs, once stored at −80 °C, were analyzed collectively in a single batch and underwent automated analysis. Additionally, after completing analysis of all the samples, the initial five samples analyzed were assessed again, revealing values exhibiting less than a 10% variation.

### 2.7. Statistical Analysis

The distribution of each variable was evaluated using the Shapiro–Wilk test, revealing a *p*-value < 0.05 for all parameters. Additionally, QQ plots indicated that the data did not conform closely to a diagonal line. Consequently, the data were deemed non-normally distributed, prompting the adoption of a nonparametric approach for analysis. Group comparisons were conducted using the Mann–Whitney test to evaluate differences in each variable. The results were presented as median and range.

To assess stability, the assay’s imprecision was measured using the intra-assay coefficient of variation (Intra-CV). At each measurement point, the loss and recovery of cystatin C, ammonia, and bicarbonate were calculated as percentages relative to the initial analysis (T0, set as 100% of each analyte concentration in the saliva sample). This calculation was carried out using the formula: (T − T0) × 100/T0. Consequently, cystatin C, ammonia, and bicarbonate were deemed stable if changes observed in the stored samples did not exceed the acceptable significant change limit (SCL) for the assay. The SCL was defined as SCL = 100% ± 2 × Intra-CV [24]. To determine the statistical significance of the percentage changes observed in analyte levels over time at different temperatures, a two-way ANOVA test of repeated measures, coupled with Dunnett’s multiple comparison tests, was employed. Changes surpassing the Significant Change Limit (SCL) and showing significant deviations from the baseline (T0) were considered indicative of unacceptable stability under the specified storage conditions.

All the statistical calculations were made using commercially available software (GraphPad Prism 9, GraphPad Software, San Diego, CA, USA).

## 3. Results

### 3.1. Analytical Validation of Cystatin C, Ammonia and Bicarbonate Assays

The cystatin C assay exhibited mean intra- and inter-assay CVs of 3.2 and 4.5%, respectively. The ammonia assay showed mean intra- and inter-assay CVs of 3.2 and 9.7%, while the bicarbonate assay displayed mean intra- and inter-assay CVs of 3.9 and 11.5%.

**Figure 1:**
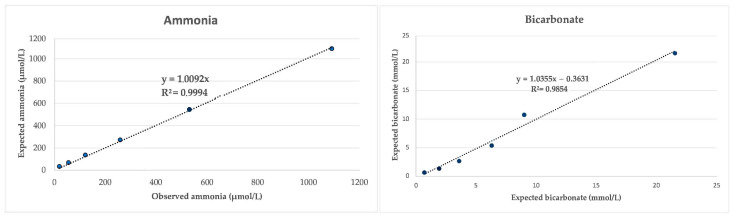
Linearity under dilution of the ammonia and bicarbonate assays.

The investigation into accuracy revealed that the serial dilution of the saliva samples exhibited linear regression equations with correlation coefficients approaching 1 for both ammonia and bicarbonate (Figure 1). In the case of cystatin C, dilution with distilled water produced interference, and accuracy was evaluated through a recovery study that showed recovery rates from 91.9 to 112.1% (Table 1). The LLQ was set at 0.24 mg/L for salivary cystatin C, 26.98 µmol/L for ammonia, and 1.15 mmol/L for bicarbonate assay. The LD of the bicarbonate assay was set at 1.51 mmol/L, while the LD of the cystatin C and ammonia assays could not be determined since all measurements with ultrapure water gave negative values.

### 3.2. Stability of Cystatin C, Ammonia, and Bicarbonate Assays

**Figure 2:**
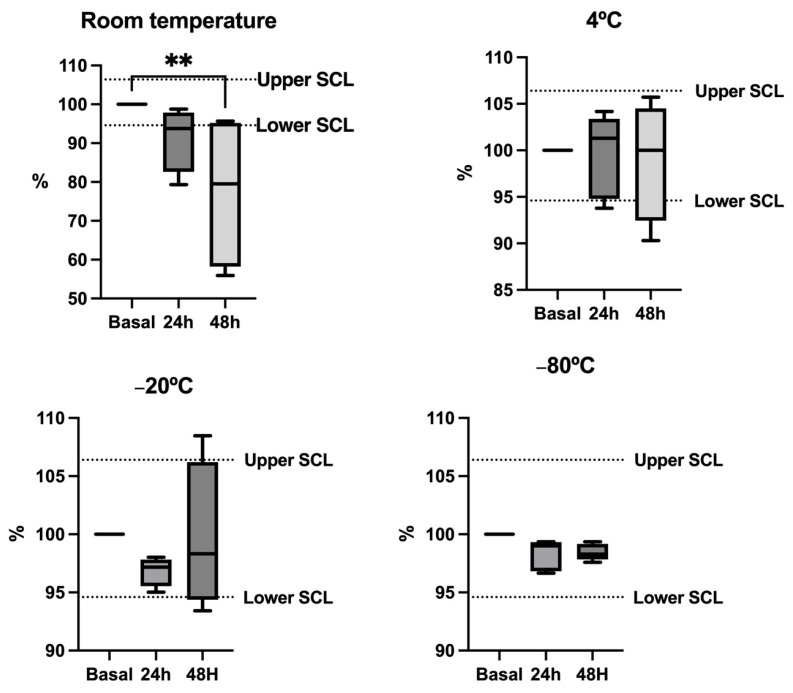
Stability plots depict the levels of cystatin C in five pig saliva samples stored at various temperatures. Results are expressed as percentages of the baseline concentration (fresh sample = 100% concentration). Each sample was stored at different temperatures and measured under fresh conditions (Baseline) after 24 and 48 h. The dotted lines represent the significant change limit (SCL) deemed acceptable for the assay, defined as SCL = 100% ± 2 × Intra-CV. The plot displays the median (line within the box), 10th and 90th percentiles (box), and the minimum and maximum values (whiskers). Asterisks denote significant differences from the baseline value (**: *p* < 0.01).

Figure 2 shows the results of the stability test for cystatin C. After 48 h, the saliva samples’ median concentrations were unstable at room temperature, showing significantly lower levels than the basal values and below the SCL. During the first 24 h, the median concentrations were close to the lower limit of the SCL, although the changes were not significant. However, cystatin C showed no significant changes in samples stored at 4 °C, at −20 °C, and −80 °C, keeping the median concentrations within the SCL at these temperatures. The lower variability was observed in samples stored at −80 °C.

**Figure 3:**
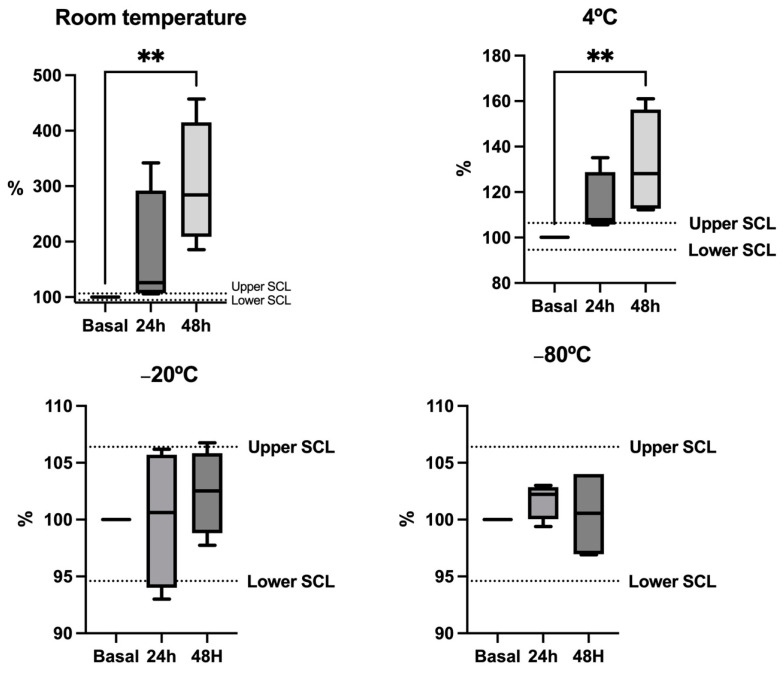
Stability plots illustrate the levels of ammonia in five pig saliva samples stored at various temperatures. The results are expressed as percentages of the baseline concentration (fresh sample = 100% concentration). Each aliquot was stored at different temperatures and measured under fresh conditions (Baseline) after 24 and 48 h. The dotted lines represent the significant change limit (SCL) deemed acceptable for the assay, defined as SCL = 100% ± 2 × Intra-CV. The plot displays the median (line within the box), 10th and 90th percentiles (box), and the minimum and maximum values (whiskers). Asterisks denote significant differences from the baseline value (**: *p* < 0.01).

Ammonia stability in pig saliva is shown in Figure 3. Median concentrations from samples stored at room temperature and 4 °C exceeded the SCL, being higher than the baseline values after 24 and 48 h, indicating significant alterations compared to the baseline values. When samples were frozen at −20 °C or −80 °C, they did not show significant changes in their median concentrations during the duration of the stability study with values within the SCL.

**Figure 4:**
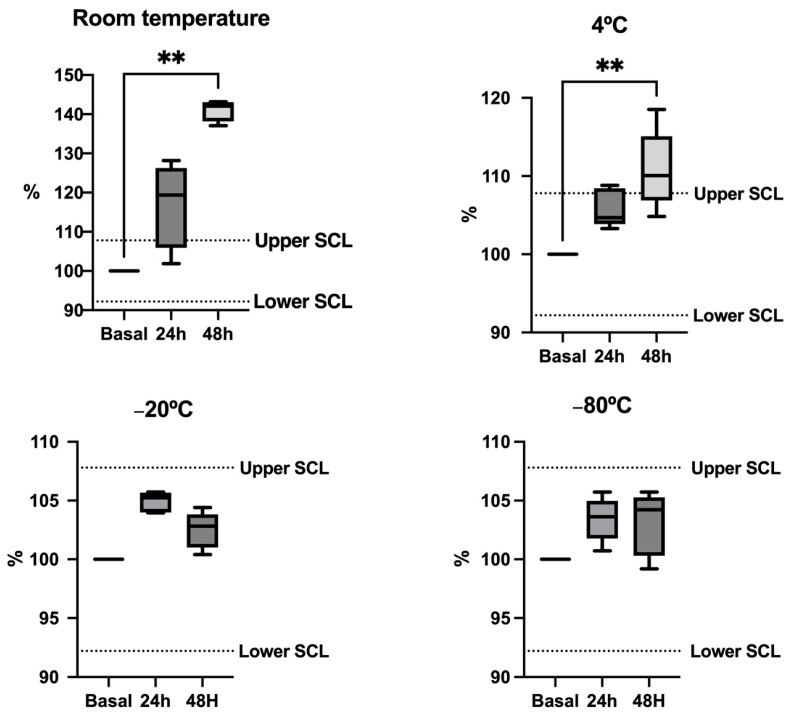
Stability plots depict the levels of bicarbonate in five pig saliva samples stored at various temperatures. Results are expressed as percentages of the baseline concentration (fresh sample = 100% concentration). Each aliquot was stored at different temperatures and measured under fresh conditions (Baseline) after 24 and 48 h. The dotted lines represent the significant change limit (SCL) deemed acceptable for the assay, defined as SCL = 100% ± 2 × Intra-CV. The plot shows the median (line within the box), 10th and 90th percentiles (box), and the minimum and maximum values (whisker). Asterisks indicate significant differences from basal (**: *p* < 0.01).

For bicarbonate, the stability data are displayed in Figure 4. Bicarbonate concentrations were higher than the SCL after 24 h when stored at room temperature, significantly increasing compared with the basal concentrations. Concentrations stable within the SCL at 24 h if stored at 4 °C but turn unstable after 48 h at this temperature with significantly higher values than the basal. In the case of frozen samples, they did not show significant changes at −20 and −80 °C, showing concentrations within the SCL in both cases.

### 3.3. Concentrations of Cystatin C, Ammonia, and Bicarbonate in Pigs with Meningitis Due to S. suis 

**Figure 5:**
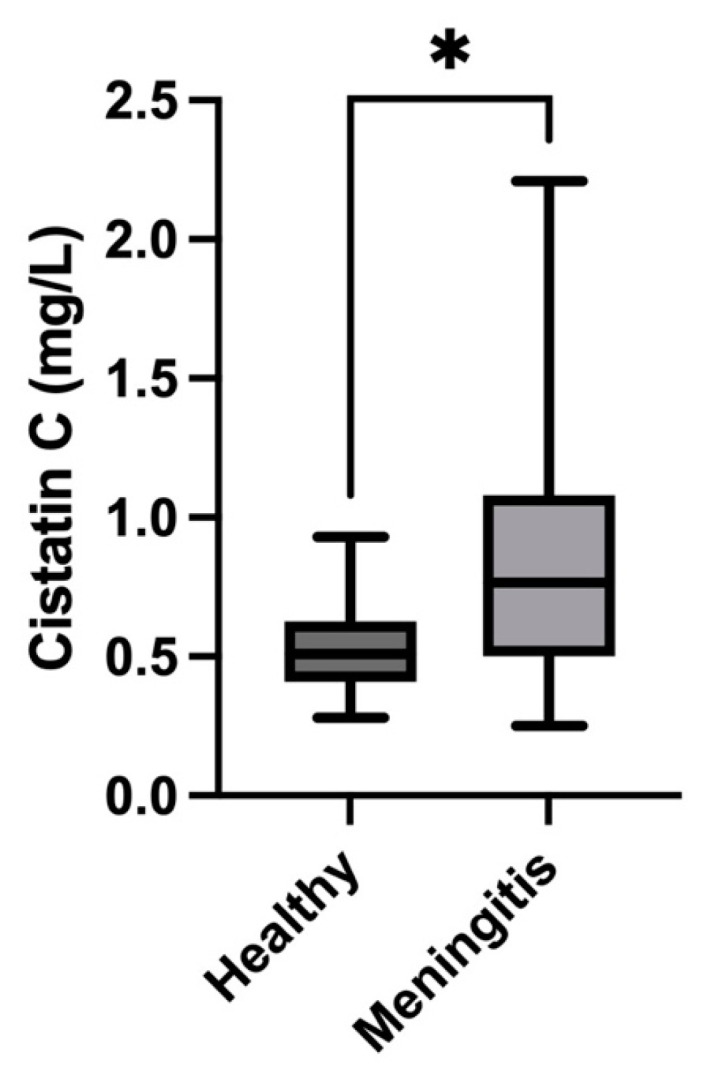
Concentrations of salivary cystatin C in pigs with meningitis due to *S. suis* compared with healthy pigs. The median values are represented by the lines, the 10–90 percentiles by the boxes, and the range by the whiskers. * *p* < 0.05.

**Figure 6:**
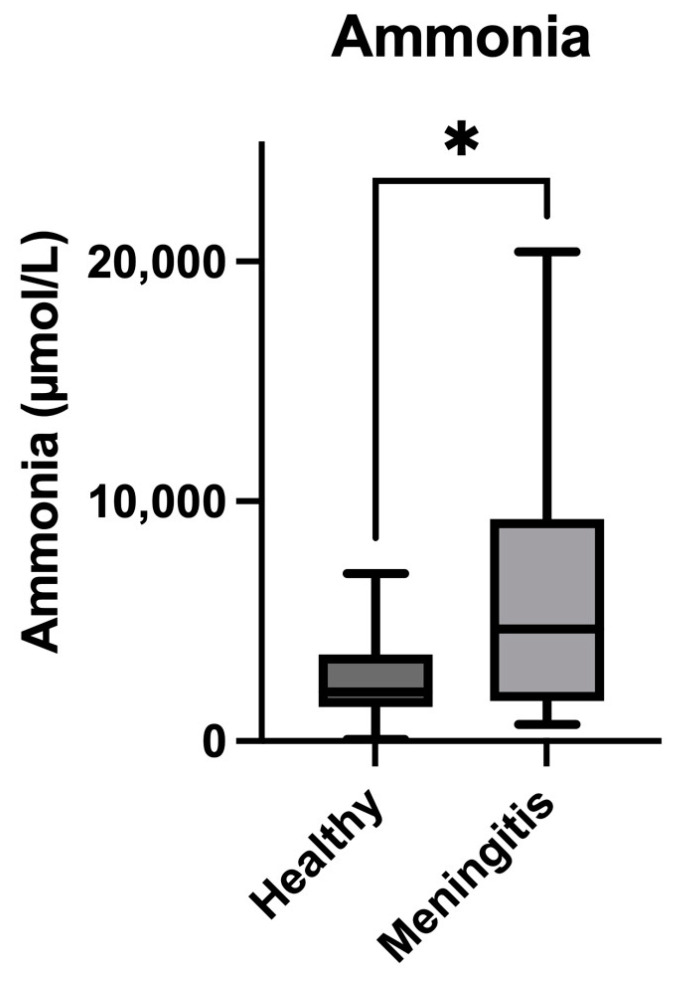
Concentrations of salivary ammonia in pigs with meningitis due to *S. suis* compared with healthy pigs. The median values are represented by the lines, the 10–90 percentiles by the boxes, and the range by the whiskers. * *p* < 0.05.

**Figure 7:**
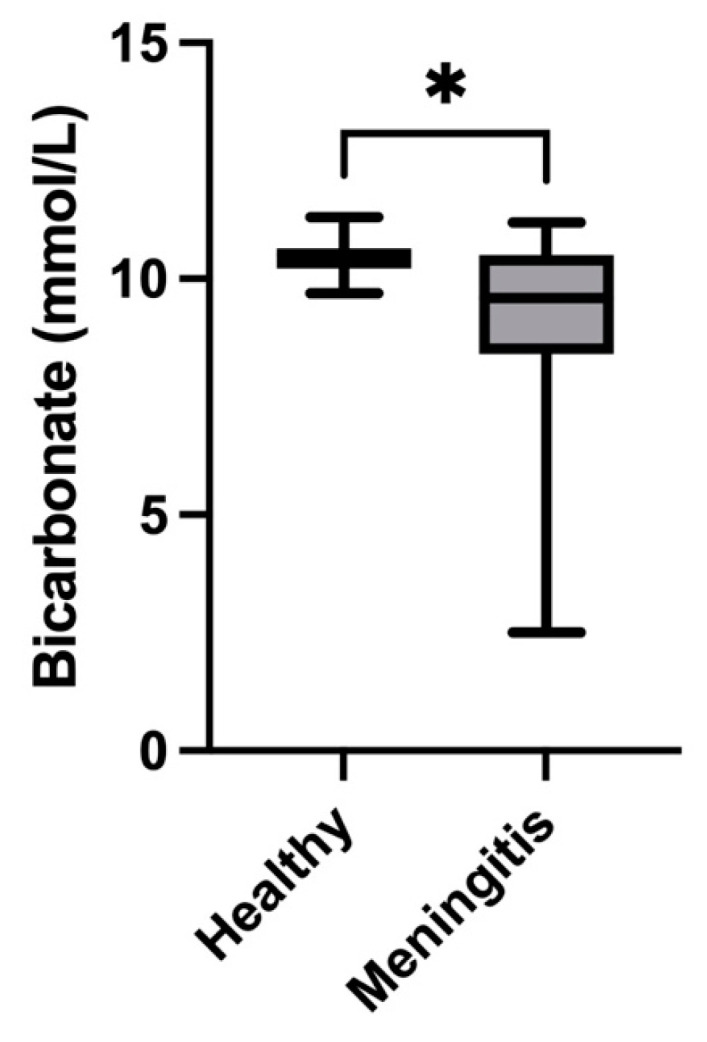
Concentrations of salivary bicarbonate in pigs with meningitis due to *S. suis* compared with healthy pigs. The median values are represented by the lines, the 10–90 percentiles by the boxes, and the range by the whiskers. * *p* < 0.05.

Salivary cystatin C concentrations were significantly higher in pigs with meningitis caused by *S. suis* (median = 0.76 mg/L; IQR = 0.5–1.08) compared with healthy pigs (median = 0.51 mg/L; IQR = 0.41–0.62) (*p* = 0.01) (Figure 5). In the case of ammonia, pigs with meningitis showed higher levels (median = 4683 µmol/L; IQR = 1671–20,414) than healthy pigs (median = 2047 µmol/L; IQR = 1425–6999) (*p* = 0.02) (Figure 6). Finally, the concentrations of bicarbonate in the saliva of pigs with meningitis were significantly decreased (median = 9.6 mmol/L, IQR = 8.4–10.5) compared with healthy pigs (median = 10.5 mmol/L, IQR = 10.2–10.6) (*p* = 0.01) (Figure 7).

## 4. Discussion

In this report, automated assays for three analytes—cystatin C, ammonia, and bicarbonate—are validated in the saliva of pigs. In addition, it is also reported that these analytes can show changes in saliva in sepsis, such as in pigs naturally infected with *S. suis*. To our knowledge, assays for the measurement of these analytes in the saliva of pigs have not been described, and the data of this report can provide a basis for future studies in which their possible applications as biomarkers could be explored.

The three assays used in this study showed adequate accuracy data, with high linearity of values after serial sample dilution in the case of ammonia and bicarbonate and high recovery rates when mixing samples of different values in the case of cystatin C. Also, they were precise according to the criteria described for the bioanalytical assay validation [25]. Based on this data, the assays could be used for the measurements of Cystatin C, ammonia, and bicarbonate in the saliva of pigs. To our knowledge, this is the first report of a specific assay for the measurement of cystatin C in the saliva of any animal species, and in the case of ammonia and bicarbonate, the validation results of this study were similar to those obtained in an analytical validation of these methods made in horse saliva [14]. In the case of ammonia and bicarbonate, the assays validated are spectrophotometric and, therefore, potentially could also be used in other animal species; however, for cystatin C, since it is an immunoturbidimetric assay that uses antibodies, a previous validation in the species to be used should be performed.

The evaluation of the stability after storage indicated that the three analytes are stable for short-term storage at −80 °C, at −20 °C and even at −4 °C in the case of cystatin C. Artifactual changes in ammonia and bicarbonate have been described in horse saliva and human saliva at room temperature [14,26]. In accordance with this data, storage at −80 °C would be recommended for these analytes.

Cystatin C belongs to the type 2 family of the cystatin superfamily, and it is ubiquitously distributed in animals and also in plants [27]. This wide diffusion and homology in its structure could explain why the antibodies of the assay used in our study, originally designed for humans, had cross-reactivity with pigs. It can inhibit the growth of bacteria, bind to the active sites of cysteine and thiol proteases, reduce their function, and decrease the production of mediators of inflammation and also ROS and NO in bacterial infection. Therefore, it has antimicrobial and immunomodulation properties [28]. Immunomodulation is specially focused on the immune innate response with modulation of cathepsin activity, antigen presentation, and synthesis of tumor necrosis factor-alpha and interleukin 10 [29].

In our report, cystatin C is increased in the saliva of pigs with *S. suis*, which is an infection condition leading to sepsis. This data agrees with the increases in this protein in sepsis and inflammation that have been detected in a previous proteomic study in the saliva of pigs [10]. This is also in line with studies in humans in which serum cystatin C concentrations are increased in patients with sepsis and can potentially act as a biomarker for early diagnosis, to assess severity, and to evaluate the prognosis of this condition [30]. However, in humans, they are also involved in other diseases such as diabetes, renal failure, tumors, and neurodegenerative diseases, so further studies should also be performed to evaluate how cystatin C can change in the saliva of other diseases different than sepsis and their possible applications [27].

In our study, there was an increase in ammonia in the saliva of pigs with *S. suis*. High concentrations of ammonia in sepsis could associated with the damage produced in this condition at the intestine and also with the increase in the metabolism of amino acids leading to ammonia production [12]. In addition, ammonia can be increased in cases of renal dysfunction [31] that can also be produced in sepsis [32]. The increase found in this report is in line with the high values reported in serum ammonia in human patients with sepsis. In humans, ammonia is also an independent risk factor for 28-day mortality, having a similar predictive value to SOFA, with the advantage of being easier to obtain considering the complexity of calculating the SOFA score and its requirement for the assessment of numerous indicators [12]. In addition, increased concentrations of ammonia in serum have been related to longer hospital stays for patients with sepsis [12]. Further studies would be of interest to evaluate the possible use of ammonia concentrations in saliva to predict mortality in *S. suis* infection.

The decrease found in our report in bicarbonate in *S. suis* infection could be related to the metabolic acidosis that is produced in sepsis; in addition, hemodynamic instability in sepsis can induce multiple organ compromise, including renal dysfunction and tissue hypoperfusion, both of which can cause decreased serum bicarbonate levels [15]. This decrease would be in line with the decreases found in serum in humans and sheep with sepsis [15,16]. Similar to ammonia, serum bicarbonate is considered a predictor of clinical outcomes in cases of sepsis, as low serum bicarbonate is associated with significantly increased in-hospital mortality [17]. The concentrations of bicarbonate obtained in our study are in the range of the values previously reported in the saliva of pigs, giving also similar values to those in humans [18].

This study should be considered as a pilot report, and more research about the possible applications of the measurements of cystatin C, ammonia, and bicarbonate in saliva as biomarkers of sepsis and inflammation, and in the case of cystatin C of other diseases such as renal failure, should be performed. In addition, the possible use of these biomarkers as prognostic factors and for treatment monitoring, as well as the possible changes in these analytes according to the severity of the disease in *S. suis* infection, should be explored. Additionally, further studies in a large population of pigs with the disease should be performed to assess the capability of these markers individually or combined to detect the infection and monitor the treatment.

Overall, this report provides the basis for the possibility of measuring these analytes in saliva in an easy and fast way and will facilitate the performance of additional studies that will help elucidate their possible practical uses and applications. These applications will have the advantages associated with saliva samplings, such as the lack of production of stress and the lack of need for qualified personnel to perform it.

## 5. Conclusions

Cystatin C, ammonia, and bicarbonate can be measured in saliva samples of pigs with adequate analytical performance, including precision and accuracy. Cystatin C and ammonia increase, and bicarbonate decreases, in the saliva of pigs with *S. suis* infection. Further studies should be performed to evaluate the potential of these analytes as biomarkers of sepsis and inflammation.

## Figures and Tables

**Table 1:** Results of the recovery assay after mixing two porcine saliva samples with different concentrations of cystatin C.

Percentage of Sample 1	Expected Concentrations	Observed Concentrations	Percentage of Sample 2	Recovery (%)
100	1.49	1.49	0	
75	1.24	1.35	25	91.9
50	0.99	0.94	50	105.3
25	0.74	0.66	75	112.1
0	0.49	0.49	100	

## Data Availability

The data presented in this study are available on request from the corresponding author.
